# Malaria Control in the Environment of the Cantonments

**Published:** 1919-01

**Authors:** 


***Malaria Control in the Environment of the Cantonments.*** By J. A. Le Prince. *Southern Medical Journal*, August, 1918.

Soon after the declaration of war with Germany it was decided to establish military cantonments and aviation fields in the southern states.

In general, five areas were considered as needing attention or control measures :

(1) A strip of land one mile wide surrounding the cantonment from which anopheles could reach the cantonment.

(2) The inhabited area between the military reservation and the nearest town or village.

(3) The town near cantonment in which the soldiers and officers would spend their furlough periods.

(4) A strip of land surrounding this town and from which anopheles could enter it.

(5) Amusement parks and other places where the enlisted men would probably be after sundown.

For the area surrounding the cities and beyond the cantonment lines surveys were made. Proper ordinances were passed by both city and county authorities to prevent the production of mosquitos in water containers, which resulted largely in the removal or elimination of water-holding vessels.

At some of the towns cases of malaria were of frequent occurrence and anopheles breeding places very numerous. Also large numbers of mechanics and laborers had recently arrived from distant points, who lived in the town and worked at the cantonments. In some instances this new influx of population contained more malaria-carriers than were originally in the city. It was necessary to prevent mosquito production at once. Therefore, as rapidly as possible, oil was applied to all natural water surfaces, pools, swamps, streams and ditches, by means of knapsack sprayers.

In the large areas surrounding the cantonments, which contain many square miles, the stream banks are often inaccessible, due to the heavy brush and green briar on the banks. The removal of brush has a marked effect in drying streams and reducing the amount of oiling necessary.

Where the streams had a fair current, oiling was done by means of drip cans and the oil film distributed by the current.

In many districts, by using a small motor truck or wagon body on which an oil tank was installed, the amount of work accomplished by the oil gang has been doubled.

At several cantonments the stream beds are of such a character that it is not practical to train the water to a narrow channel, as it would be changed by the next rainstorm; but wherever the texture of the soil allowed for training of the stream or ditch bed so as to keep the flow in a narrow channel, this work was accomplished.

In addition to the protecting of our army, in the vicinity of more than 15 of our important cities of the South, steps have been taken that will go a long way toward the eradication of malaria.

